# Sensor Data Fusion Based on Deep Learning for Computer Vision Applications and Medical Applications

**DOI:** 10.3390/s22208058

**Published:** 2022-10-21

**Authors:** Rizwan Ali Naqvi, Muhammad Arsalan, Talha Qaiser, Tariq Mahmood Khan, Imran Razzak

**Affiliations:** 1 School of Intelligent Mechatronics Engineering, Sejong University, Seoul 05006, Korea; 2 Division of Electronics and Electrical Engineering, Dongguk University, 30 Pildong-ro 1-gil, Jung-gu, Seoul 04620, Korea; 3 Department of Computer Science, University of Warwick, Coventry CV4 7AL, UK; 4 School of Computer Science and Engineering, University of New South Wales, Sydney 1466, Australia

Sensor fusion is the process of merging data from many sources, such as radar, lidar and camera sensors, to provide less uncertain information compared to the information collected from single source. Data fusion, on the other hand, is a process in which multiple data sources increase the measurement reliability, range, and accuracy. Different measuring principles are also used to confirm detected objects. The combined term sensor data fusion is defined as the gathering of data that individual sensors functioning independently cannot provide. It combines the advantages of many sensors and measurement techniques in an efficient manner. A wide range of emerging applications in computer vision, biometrics, video surveillance, image compression and restoration, medical image analysis, computer-aided diagnosis, etc., have resulted from the extensive use of various sensors, including visible light sensors, near-infrared (NIR) sensors, thermal camera sensors, fundus cameras, H&E stains, endoscopies, OCT cameras, and magnetic resonance imaging sensors. Sensor fusion-based methods give us the ability to utilize these sensor data and adjust industrial strategies to improve operations while increasing efficiency. High-quality and real-time perception mechanisms are necessary in order to obtain high accuracy when deploying computer vision and deep learning applications. However, there are few studies on information processing and merging sensor and data fusion and fusion architecture for cooperative perception and risk assessment for computer vision and medical applications in the literature.

The performance of computer vision technology still faces challenges due to the impact of various external environmental factors. Moreover, other challenges in this area also need to be solved or improved. In order to ensure greater accuracy, current systems have sought to combine data from numerous sensors based on deep learning techniques. This Special Issue in *Sensors* entitled *Sensor Data Fusion Based on Deep Learning for Computer Vision and Medical Applications* aims to explore high-caliber, cutting-edge research proposals in areas including multiple-approach fusion, spoof detection, image detection, localization, classification, and segmentation by deep learning to tackle challenging problems in computer vision and medical applications. To this end, while numerous manuscripts were received for consideration, only 10 high-caliber and original manuscripts were selected after an extensive peer-review process. Although some provide interesting suggestions for computer vision and medical applications, most of the proposed methodologies discussed in this Special Issue are focused on applications in sensor data fusion.

## 1. Relevant Contributions Related to Computer Vision Applications

The significant studies included in this Special Issue that deal with sensors as the primary technology for developing computer vision applications can be summarized as follows:

In [1], a 3DMesh-Group Activity Recognition (GAR) approach is proposed to observe multi-person actions via a picture, a video frame, or the inertial, environmental, physiological, and sensor data of a scene. The proposed methodology only utilizes a body center heat map, a camera map, and a mesh parameter map instead of a noisy and complex 3D skeleton of each person as an input frame. 3DMesh-GAR comprises three stages. Stage I involves a 3D mesh creation network with the purpose of creating 3D body meshes through input image frames with no additional computational expense in scenes with many people and significant occlusion. GAR is a valuable tool for monitoring the functional, cognitive, and physical health of patients in their homes or in hospitals as the elderly population increases. Recurrent neural networks (RNNs) have been used in research to recognize individual and group behaviors across time.

The work in [2] explored coral’s importance for marine ecosystems and proposed a convolutional neural network (CNN)-based model termed DeeperLabC to enhance the performance efficacy of underwater monitoring. The proposed DeeperLabC is one of the most effective techniques for automatically examining coral in underwater photos with the help of semantic segmentation to monitor marine pollution brought on by global environmental changes. In addition, DeeperLabC was trained on the gathered dataset CoralS. DeeperLabC is a simplified and modified version of the deeper lab model with encoder–decoder architecture for the semantic segmentation of corals. ResNet34 was employed as a backbone architecture for further extraction of coral characteristics from single-channel coral images and to carry out semantic segmentation. The simulation results indicate that DeeperLabC outperformed the baseline model in a semantic segmentation task, achieving coral segmentation with an F1 score value of 97.10% and a mean intersection over union (IoU) value of 93.90%.

The study in [3] presents the novel idea of linking visual sentiment with human activity analysis and shows how images of natural catastrophes affect people’s feelings and perceptions. This study examines complex issues, including human emotions and corresponding bodily behaviors in response to disaster-associated images gathered from social media platforms. More than 3500 images with three different sets of tags were collected, producing four separate datasets of various sentiments and related human activity hierarchies. In the proposed framework, datasets are collected from different social media, and the extraneous images are removed before the second phase. More than 3500 images with three different sets of tags were collected, producing four separate datasets of various sentiments and related human activity hierarchies. Secondly, a YOLO-based human count tracker was utilized that allows for the monitoring of individuals in an obscured environment with fewer identity transitions and gives a precise count of the number of people at risk from visual content in an emergency.

The authors in [4] focus on the deep feature extraction and classification of android malware images. Malware is any program designed for a harmful purpose (malicious software). Malware typically involves disrupting routine operations, stealing sensitive data, displaying unwelcome advertising, or secretly taking control of the user’s device. The proposed Summing of neurAl aRchitecture and VisualizatiOn Technology for Android Malware identification (SARVOTAM) first converts the malware’s non-intuitive features into high-quality fingerprint images to extract valuable information. To reduce the cost of feature engineering and domain knowledge, a modified CNN is used to automatically remove rich features from the imaged malware. A total of fifteen distinct combinations of the picture portions were utilized to identify and classify android malware. Secondly, the DREBIN dataset was used for the experiment. Furthermore, it was observed that the CNN-SVM model outperformed the CNN-KNN, the CNN-RF, and the original CNN. The simulation results show that the proposed method achieves an accuracy of 92.59% when using android certificates and manifest malware images.

The recognition of handwritten characters is one of the most challenging and exciting research fields in pattern recognition and computer vision. Moreover, the significance of differences in writing styles and cursive text, as well as the resemblance of different characters, make it more challenging. To deal with these challenges, the work in [5] proposed a CNN model to recognize Pashto’s handwritten characters (PHC). The proposed CNN-based model can identify and categorize handwritten Pashto characters. The proposed model has the benefit of being adaptable to character recognition tasks such as handwritten character recognition and optical character recognition for almost any accessible language, such as English, Arabic, Hangul, etc. In the beginning, a 44-character Pashto handwritten character dataset termed “Poha” is constructed. The experimental results demonstrate that the proposed model outperformed the baseline models in terms of accuracy. The accuracy of the proposed CNN model is 99.64 percent for PHC recognition.

## 2. Relevant Contributions Related to Medical Applications

The following are some of the significant studies that deal with sensors as the main method for developing medical applications that are featured in this Special Issue.

In medical cancer treatment, lung cancer has developed into a hazardous condition with a low chance of survival. Early detection and appropriate care can improve survival chances. Analyzing medical images with a deep learning computer-aided diagnostic system has been shown to yield significant improvements in a variety of medical applications. To this end, the research in [6] presents a comparative study of different CNN-based models, such as eNet, AlexNet, VGG16, ResNet-50, and Inception-V1, where the authors compare the accuracy of these algorithms for lung cancer detection using the publicly available LUNA16 dataset. The simulation results illustrate that the CNN-based AlexNet architecture with the SGD optimizer has the highest accuracy with a precision of 97.42%.

The work proposed in [7] demonstrates that Alzheimer’s disease (AD) is one of the most progressive and irreversible degenerative cerebral illnesses characterized by psychological disability. According to the report, around 300 million people worldwide are expected to die from Alzheimer’s disease by 2050. Developing a particular medication that can treat or cure AD and prolong the patient’s life is still a challenging task. The progression of AD can be slow with simple medical therapies if it is diagnosed in its early stages. Moreover, early diagnosis is also beneficial for people with endurance disorders. The proposed methodology in this work uses recurrent neural networks to forecast this disease. This model predicts the biomarkers for patients diagnosed with AD after 6, 18, 12, 21, 24, and 36 months. Thereafter, these anticipated biomarkers will go through layers of a neural network that are completely coupled. The proposed model shows an accuracy of 88.24% with the openly available informational dataset by the Alzheimer’s Disease Neuroimaging Initiative (ADNI).

Identifying and categorizing skin cancer and the variety of skin textures and injuries is challenging. It takes time and effort to manually detect skin lesions from dermoscopy images. In [8], an automated deep learning-based system for multiclass skin lesion classification is proposed, where deep learning feature fusion and extreme learning machine approaches are utilized. The proposed multiclass lesion classification approach comprises five steps, including image acquisition, deep learning feature extraction using transfer learning, contrast enhancement, best feature selection using a hybrid whale optimization and mutual entropy information (EMI) approach, the fusion of selected features using a modified canonical correlation-based method, and extreme learning machine-based classification. Two publicly available datasets, HAM10000 and ISIC2018, are used to validate the model efficacy. For both datasets, 93.40 and 94.36 percent accuracy was attained, respectively. As a result, the lesion region’s quality is improved by the contrast enhancement phase, followed by the extraction of the more pertinent characteristics and accuracy of the classification.

The work proposed in [9] explores the frequent and deadly condition of diabetic retinopathy (DR), which affects eyesight and can result in blindness or total vision loss. The early warning signals that might result in complete vision loss in the eyes are called microaneurysms (MAs). These MAs have an almost round shape, a darkish tint, and a small size that makes them invisible to ophthalmologists during manual examinations. It is challenging to identify and classify microaneurysms since they might have a variety of characteristics on fundus imaging, such as texture, color, and size. According to the research, many specialists claim that early detection of DR can rescue over 90% of diabetes patients. To solve the aforementioned challenges, this work pre-trained a CNN model using a hybrid feature-embedding technique that has been proposed for the early detection of MA. Two publicly accessible datasets, E-Ophtha and DIARETDB1, were used to test the performance of the suggested technique. The results show that the proposed framework obtained classification accuracies of 96% and 94% for E-Ophtha and DIARETDB1, respectively. DIARETDB1 was utilized to gather fundus patches from 70 shots intended to capture MA signals for the training phase. Therefore, compared to DIARETDB1, the E-Ophtha dataset’s overall accuracy was increased by 0.2%, and its specificity was increased by 0.01%.

Bluetooth low energy (BLE)-based contact tracing is a crucial component of COVID-19 mitigation. In recent years, many applications have been introduced to monitor and manage the viral distribution of COVID-19. Most of these applications employ BLE broadcasts, which are used to identify vulnerable and infected people near an infected person and have a small range. The authors in [10] utilize BLE technology to deal with COVID-19 hazards in academic buildings and indoor classroom settings. They use a sigmoid-based model for epidemics with various distance thresholds to categorize contact data as low or high risk based on characteristics such as contact time. Moreover, ML classifiers such as support vector machines, decision trees, linear discriminant analyses, k-nearest neighbors, and logistic regression are used to classify a person as high or low risk based on labeled data of received signal strength indicators (RSSI) and distance. The analysis of the epidemic model reveals that at the 50 cm threshold for the linear epidemic model for the indoor dataset, all students are at risk; however, at the same time, no student is in danger when using the sigmoid epidemic model.
